# Variations and National Perspectives on Evaluation and Management of Ventilator-Associated Pneumonia in Neonatal Intensive Care Units: An In-Depth Survey Analysis

**DOI:** 10.7759/cureus.64944

**Published:** 2024-07-19

**Authors:** Irfan Shehzad, Muppala Raju, Shabih Manzar, Gueorgui Dubrocq, Malvika Sagar, Niraj Vora

**Affiliations:** 1 Neonatalology, Christus Children’s Hospital, San Antonio, USA; 2 Neonatology, Baylor Scott & White Health, Temple, USA; 3 Neonatology, Louisiana State University Health Science Center, Shreveport, USA; 4 Pediatric Infectious Diseases, Baylor Scott & White Health, Temple, USA; 5 Pediatric Pulmonary, Baylor Scott & White Health, Temple, USA

**Keywords:** practice variations, ventilator-associated pneumonia (vap), invasive mechanical ventilation, preterm neonates, neonatal intensive care unit (nicu)

## Abstract

Introduction

Infants in the neonatal intensive care unit (NICU) are vulnerable to ventilator-associated pneumonia (VAP), which increases their morbidity and mortality. There is a significant overlap of clinical features of neonatal VAP with other pulmonary pathologies, particularly in preterm infants, which can make the definitive diagnosis and management of VAP challenging.

Objective

Our study surveyed NICU providers across the United States to understand the perspectives and variations in neonatal VAP diagnostic and management practices.

Methods

The REDCap survey was distributed to the actively practicing members of the Section on Neonatal-Perinatal Medicine (SoNPM) of the American Academy of Pediatrics (AAP). We used descriptive statistics to analyze the data from the respondents.

Results

Of 254 respondents, the majority (86.6%, 220) were neonatologists and had a relatively even geographical distribution. Most (75.9%, 193) stated that they would perform a gram stain and respiratory culture as part of a sepsis workup irrespective of the patient’s duration on invasive mechanical ventilation (IMV); 224 (88.2%) of providers preferred the endotracheal aspiration (ETA) technique to collect specimens. In cases where a positive respiratory culture was present, VAP (52.4%, 133) was the predominantly assigned diagnosis, followed by pneumonia (27.2%, 69) and ventilator-associated tracheitis (VAT) (9.8%, 25). Respondents reported a prescription of intravenous gentamicin (70%, 178) and vancomycin (41%, 105) as the initial empiric antibiotic drugs, pending final respiratory culture results. Most respondents (55.5%, 141) opted for seven days of antibiotics duration to treat VAP. The reported intra-departmental variation among colleagues in acquiring respiratory cultures and prescribing antibiotics for VAP was 48.8% (124) and 37.4% (95), respectively, with slightly more than half (53.5%, 136) of providers reporting having VAP prevention guidelines in their units.

Conclusion

The survey study revealed inconsistencies in the investigation, diagnostic nomenclature, choice of antibiotic, and treatment duration for neonatal VAP. Consequently, there is a pressing need for further research to establish a clear definition and evidence-based criteria for VAP.

## Introduction

Neonatal ventilator-associated pneumonia (VAP) is generally referred to as a bacterial infection of the lungs in an intubated patient, and it is the second most common healthcare-associated infection in the neonatal intensive care unit (NICU) [1,2]. It occurs at a rate of 10.9 per 1,000 ventilator days and significantly impacts neonatal morbidity and mortality [3]. The reported VAP-associated case fatality rate is around 12-16.4% [4,5]. Ralitsa et al. estimated an average increase in healthcare costs of $2063 (€1918) and longer hospital length of stay by 14 days for a patient with VAP compared to without VAP [6]. Infants on respiratory support in the NICU, especially those born premature and requiring prolonged hospitalization, are at a higher risk for VAP due to various factors such as anatomic underdevelopment, repeated airway instrumentation, dysbiosis, and limited innate and adaptive immunity [1]. Specific risk factors for VAP in the NICU include prematurity, low birth weight, parenteral nutrition, prolonged tracheal intubation, re-intubation, invasive mechanical ventilation (IMV) for at least 48 hours or more, blood transfusion, gastroesophageal reflux, and bronchopulmonary dysplasia (BPD) [7].

Establishing an accurate diagnosis of VAP in the NICU is challenging due to the varied practice of specialists, a lack of a gold standard case definition, and the significant overlap of its features with other neonatal pulmonary pathologies, such as respiratory distress syndrome (RDS), lung edema, or BPD. The most commonly used diagnostic criteria for neonatal VAP involve a combination of clinical, radiographic, and laboratory findings, according to the Centers for Disease Control and Prevention (CDC) guidelines for children younger than one year [8]. However, these criteria are not always specific or relevant for NICU patients with respiratory failure, thus leading to variability in workup and treatment among clinicians [9]. Our study surveyed NICU providers across the US to understand the perspectives and variations in neonatal VAP diagnostic and management practices.

## Materials and methods

A cross-sectional survey of 25 multiple-choice questions (see the Appendices) was created using a REDCap (Research Electronic Data Capture, v9.1.0) tool approved by Baylor Scott & White Health Institutional Review Board in Temple, Texas. Baylor pediatrics research team contacted, distributed, and collected data using the electronic mailing list software (listserv). 

The survey included questions on providers’ demographics, practicing regions of the US, institution characteristics, NICU level, individual opinion of the providers, practice variation in the performance of routine surveillance microbiological cultures for respiratory infection or colonization, clinical symptoms/signs of suspected VAP, type of sampling technique in the acquisition and processing of respiratory gram stain/microbiological culture, interpretation of the results of respiratory gram stain/microbiological culture, and utilization of chest X-ray and laboratory criteria in the diagnosis of VAP. The study also assessed the provider differences in administering the empiric antibiotic to treat suspected respiratory infection pending respiratory cultures, total antibiotic treatment duration in confirmed VAP cases, providers' approach for subspecialty consultation, and utilization of center-specific VAP prevention guidelines.

The survey was distributed via email to the practicing members (neonatologists, NICU fellows, and advanced practice providers) of the Section on Neonatal-Perinatal Medicine (SoNPM) of the American Academy of Pediatrics (AAP) in August 2022, with a reminder email sent on September 2022. Final data was analyzed using the information from REDCap. The respondents’ identity was maintained anonymous. Participation was voluntary, and no incentive was provided. According to the AAP email list software and the SoNPM directory, approximately 4,000 neonatal providers received the survey. We used descriptive statistics using REDCap statistics and plots to analyze the data from the respondents and presented them as percentages. We used 254 (total responses received) as a common denominator for estimating percentages.

## Results

**Figure 1:**
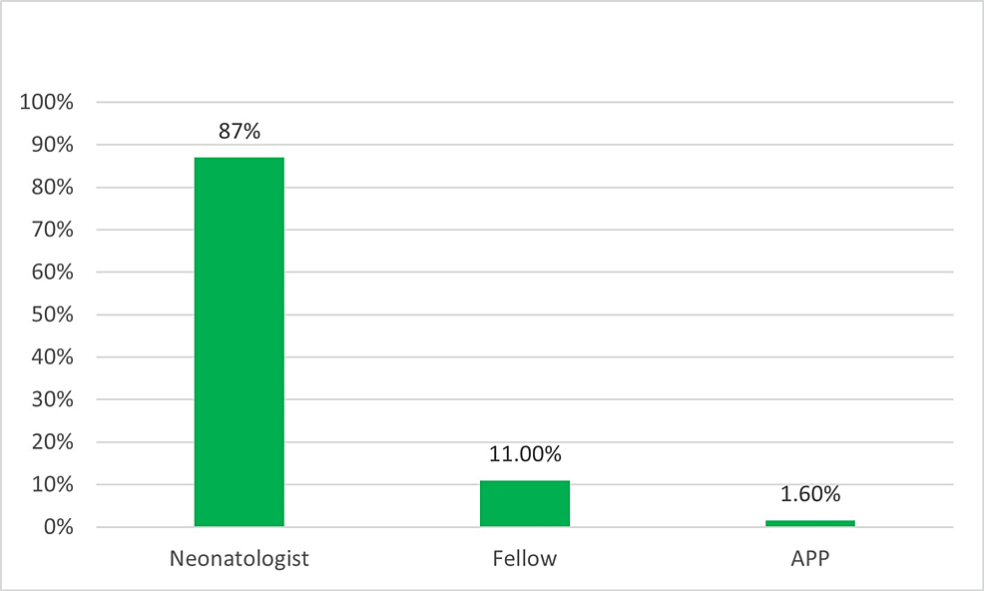
Providers

**Figure 2:**
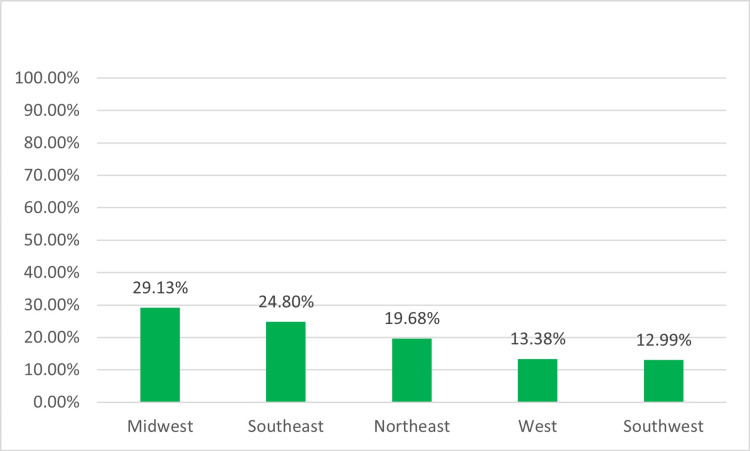
Regions of US

**Figure 3:**
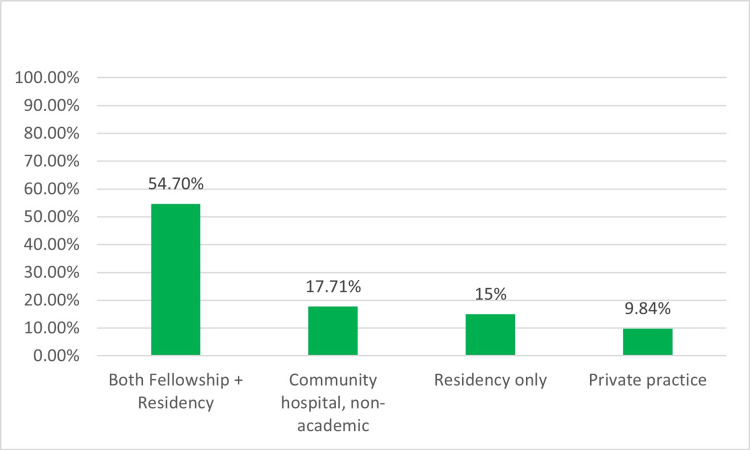
Practice type

**Figure 4:**
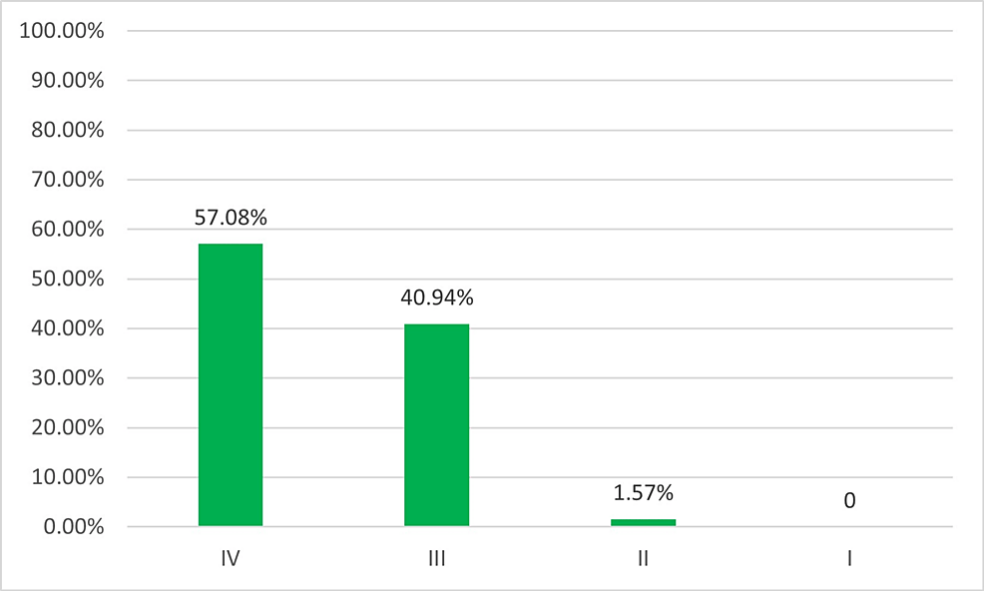
Level of NICU NICU, neonatal intensive care unit

Out of the approximately 4,000 members within SoNPM, 254 neonatal-perinatal providers participated in the survey, resulting in a response rate of 6.3% (254/4,000). Most respondents (86.6%, 220) were neonatologists (Figure 1). Participants were evenly dispersed throughout various regions of the US (Figure 2). Additionally, 69.7% (178) of participants were employed in academic centers with both pediatric residency and neonatal-perinatal medicine fellowship programs or at least a pediatric residency program (Figure 3), 98.0% (249) were affiliated with levels III and IV NICUs (Figure 4), and 79.5% (202) were managing patients in units with more than 30 beds. 

**Figure 5:**
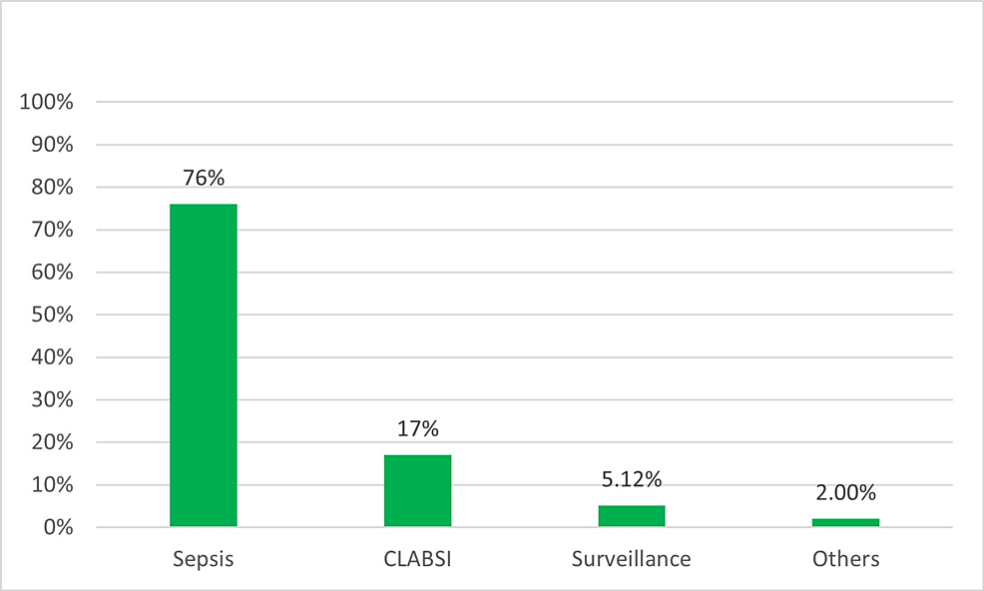
Reasons for ordering respiratory culture CLABSI, central line-associated bloodstream infection

Most providers (75.9%, 193) stated that they would perform a gram stain and respiratory culture as part of a sepsis workup, irrespective of the patient’s days on IMV. Only 5.1% (13) would perform routine surveillance respiratory cultures, and 16.9% (43) would order a respiratory culture to explore an alternative source of bloodstream infection in patients with a central line to decrease the incidence of primary central line-associated bloodstream infection (CLABSI) (Figure 5). The majority (88.2%, 224) of providers reported using endotracheal aspiration (ETA) as the most preferred technique to collect specimens, followed by bronchoscopic bronchoalveolar lavage (6.3%, 16) and non-bronchoscopic bronchoalveolar lavage (5.5%, 14). Only 0.4% (1) of providers used the non-bronchoscopic-protected specimen brush technique. Only 8.3% (21) of providers reported not obtaining respiratory cultures in the study.

**Figure 6:**
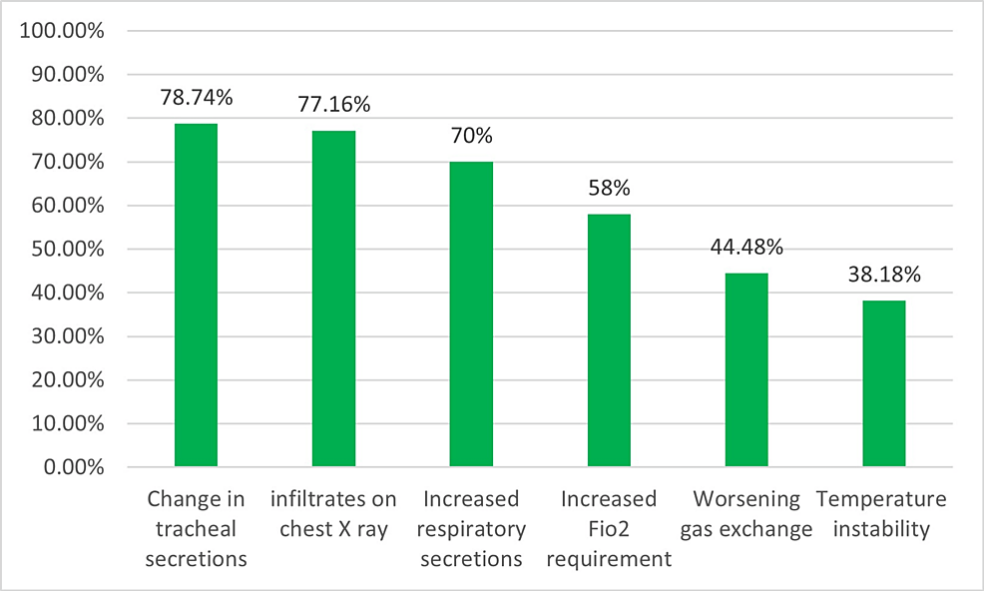
Circumstances to consider VAP VAP, ventilator-associated pneumonia

**Figure 7:**
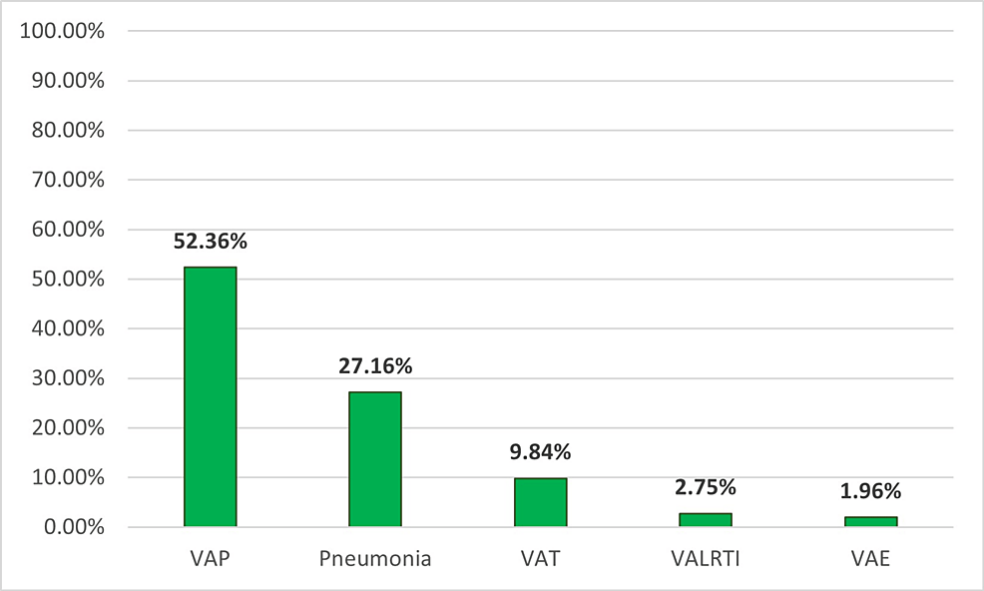
Assigned diagnosis VAP: ventilator-associated pneumonia; VAT: ventilator-associated tracheitis; VALRTI: ventilator-associated lower respiratory tract infection; VAE: ventilator-associated events

Change in the character/purulence of tracheal secretions (78.7%, 200), increase in infiltrates on chest X-ray (77.2%, 196), increase in respiratory secretions/suctioning requirement (70%, 178), increase in FiO_2_ requirement (58%, 147), and worsening of gas exchanges (44.48%, 113) prompted obtaining respiratory culture even in patients with other underlying pulmonary pathologies such as RDS, BPD, or pulmonary edema (Figure 6). The most frequently isolated bacteria were Klebsiella pneumonia (61%, 155), followed by Coagulase-negative staphylococcus aureus (CONS) (35.8%, 91), followed closely by Pseudomonas aeruginosa (33.8%, 86). In cases where a positive respiratory culture was present, VAP was the predominantly assigned diagnosis (52.4%, 133), followed by pneumonia (27.2%, 69), ventilator-associated tracheitis (VAT) (9.8%, 25), ventilator-associated lower respiratory tract infection (VALRTI) (2.75%, 7), and only 1.96% (5) as ventilator-associated events (VAE) (Figure 7).

**Figure 8:**
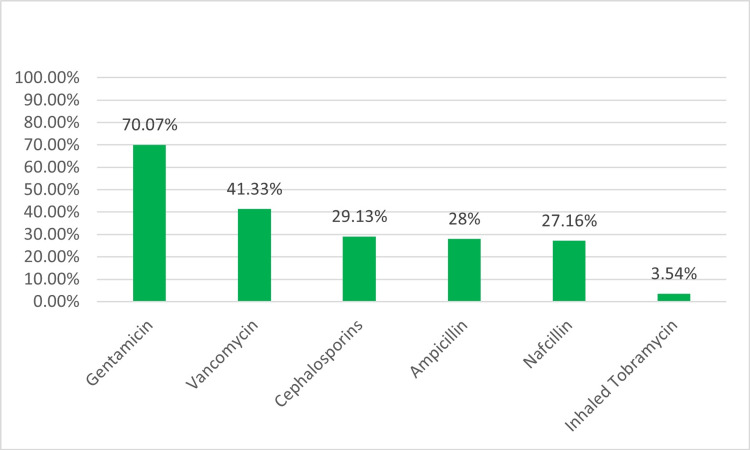
Type of antibiotics

When deciding to treat patients with VAP with antibiotics, only a minority (31.5%, 80) stated that they consider the bacterial load on quantitative or semiquantitative analysis. In comparison, the majority (70.4%, 179) responded that they would also consider the type of bacteria. Respondent-reported initial empiric drugs are IV gentamicin (70.07%, 178) and IV vancomycin (41.3%, 105), pending final respiratory culture results. Cephalosporins (29.13%, 74) were identified as the third most prescribed agent, followed by ampicillin (28%, 71) and nafcillin (27.16%, 69) as the fourth and fifth most prescribed drugs (Figure 8).

**Figure 9:**
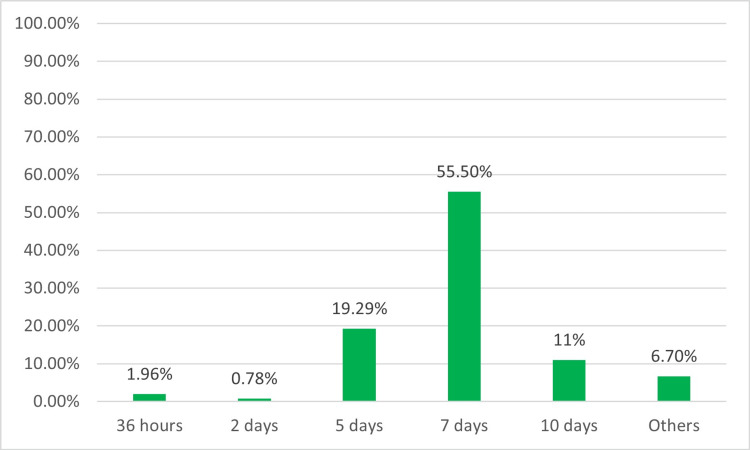
Duration of treatment

**Figure 10:**
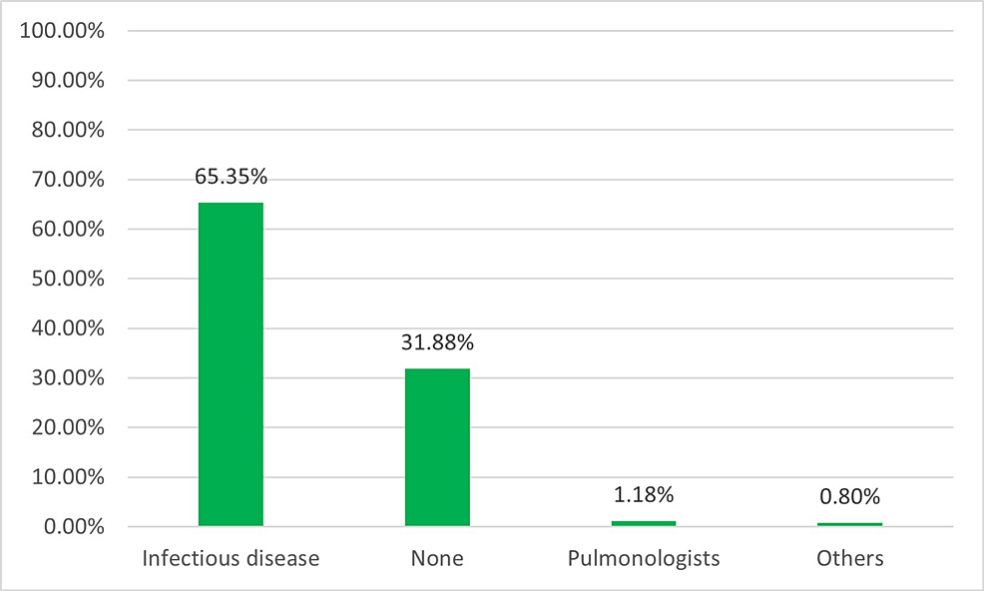
Consultation

The duration of treatment for a positive respiratory culture varied among participants. The majority (55.5%, 141) of them opted for seven days, while 19.29% (49) chose five days, and only 11.4% (29) chose 10 days (Figure 9). When we inquired about the differences in the acquisition of respiratory culture and prescription of antibiotics between colleagues in local practice, we also found intra-departmental practice variation of 48.8% (124) and 37.4% (95), respectively. Slightly more than half (53.5%, 136) of the providers reported having guidelines for VAP prevention in their units, while 45.6% (116) were either uncertain or needed policies. Regarding seeking consultation, most providers (65.3%, 166) would consult infectious disease specialists for treatment recommendations (Figure 10). 

## Discussion

In the last two decades, the survival rate of preterm infants has significantly improved due to IMV. However, IMV can lead to both short- and long-term complications, such as air leaks, interstitial emphysema, and BPD [10,11]. Neonatal VAP, a severe complication of IMV, has not received much attention in neonatal literature despite its high morbidity and mortality rates. Currently, there is no universally accepted definition of neonatal VAP. However, the most used formal criteria are derived from the CDC guidelines (Table 1) and constellate clinical, radiological, and laboratory findings [8].

**Table 1:** CDC diagnostic criteria for VAP (Adopted from NHSN, CDC. Pneumonia (VAP and non-ventilator-associated pneumonia (PNEU)) event [8]). CDC, Centers for Disease Control and Prevention; NHSN, National Healthcare Safety Network; VAP, ventilator-associated pneumonia

Required
1. Worsening gas exchange (identified based on desaturations, need for increasing ventilator settings, and/or a rising FiO_2_ requirement) AND
2. Radiographic evidence of a new or worsening focal infiltrate, consolidation, cavitation, or pneumatocele
With at least three of these findings
• Temperature instability
• Leukopenia (WBC <4,000/µL (<4×10^9^/L)) or leukocytosis (WBC >15,000/µL (<15×10^9^/L)) with a left shift (>10% band forms)
• Cough
• New-onset of purulent sputum, change in character of sputum, or increased respiratory secretions requiring increased suctioning frequency
• Apnea or increased work of breathing (tachypnea, retractions, grunting, and nasal flaring)
• Wheezing, rales, or rhonchi
• Bradycardia (<100 beats/min) or tachycardia (>170 beats/min)

Clinical criteria incorporated into VAP guidelines are nonspecific, and their sensitivity and specificity relative to the underlying pathology in NICU patients are poor [10]. They may overestimate the incidence of VAP if used in isolation. Our survey respondents' most reported clinical findings (in order of decreasing frequency) were purulence of tracheal secretions, increase in respiratory secretions, increase in FiO_2_ requirement, worsening of gas exchanges, and temperature instability. It is essential to mention that increased work of breathing, tachypnoea, and apnea may not be apparent in a ventilator-dependent patient, especially if heavily sedated, and interpretation of pulmonary sounds on auscultation is subjective in infants. Despite guidance surrounding the use of clinical criteria, providers have yet to gain consensus regarding those criteria considered for diagnosis and, therefore, need to be interpreted with care.

It is vital to understand the microbiology of VAP. Newborns can be exposed to upper respiratory tract microbiome bacteria, which can affect their lower respiratory tract. The airway microbiome can originate from the patient's flora, such as bacterial overgrowth in oral secretions, enteric microbes from refluxed gastric fluids, or the external surrounding hospital environment, including equipment and caretakers' exposure [11,12]. Usually, a combination of physical, cellular, and chemical defenses works together to keep the lower respiratory tract free of harmful microbes. However, due to immature immune systems, immaturity in anatomy, damage to lung tissue, dysbiosis that favors pathogenic species, and physical barriers posed by an endotracheal tube (ETT), pathogenic bacteria can become trapped in the lower airways [13]. Once there, these bacteria can multiply, cross tissue barriers, trigger an inflammatory cascade, and cause VAP. Gram-negative bacteria such as Klebsiella, Pseudomonas, Escherichia coli, and Enterobacter are the most isolated organisms causing VAP in NICUs, with a reported prevalence between 60% and 97% [14,15]. However, some studies have reported that gram-positive bacteria have become increasingly common, with Staphylococcus aureus being the predominant isolate [16]. Pathogens such as Pseudomonas and Acinetobacter species and methicillin-resistant strains of Staphylococcus aureus are much more common after prior antibiotic treatment, prolonged hospitalization, or mechanical ventilation [17]. Our survey asked respondents to identify the most common organism causing VAP in their unit based on respiratory culture. Responses were consistent with other studies. The organism most frequently reported by respondents were Klebsiella pneumonia (59%), followed by CONS (35%), Enterobacteriaceae (34.6%), Pseudomonas aeruginosa (33.8%), methicillin-resistant staphylococcus aureus (26.3), Stenotrophomonas (24%), Escherichia coli (25%), Streptococcus (14.5%), Hemophilus (4.3%), Moraxella (2.7%), Ureaplasma (2.3%), and Proteus (2.3%). 

Bacteremia, the presence of bacteria in the bloodstream, can occur alongside pneumonia. Patients suspected of having VAP usually undergo blood culture testing before initiating empiric antibiotic therapy. However, due to its low yield, this method is often unreliable in identifying the causative organism for VAP [1]. It is essential to note that the respiratory tract is not a sterile site, colonization is expected, and a positive respiratory tract culture from routine surveillance without clinical and radiological findings is insufficient and not reliable to diagnose VAP or to predict pathogens isolated from blood during an episode of late-onset sepsis [17,18]. According to a survey, only 5.1% of providers stated that they perform routine surveillance of respiratory cultures. This is understandable as the efficacy and utilization of such testing remain controversial.

Microbiological criteria are not mandatory to diagnose neonatal VAP, but it may be essential to consider in a clinical context. During treatment, respiratory culture and microscopic examination can aid in determining the patient's response to antibiotics [19]. However, there is a need to standardize microbiological sampling and processing. The endotracheal aspirate (ETA) technique is often used by healthcare professionals to perform gram staining and microbial sampling in suspected VAP. Still, it has limited sensitivity and specificity due to contamination [20]. While ETA can help rule out VAP, it has a high rate of false positives and can lead to unnecessary use of antibiotics. More precise microbiologic identification of lower airway pathogens can be achieved by bronchoalveolar lavage and bronchoscopic brush techniques [21]. These techniques are less likely to yield contaminated and polymicrobial specimens compared to upper airway sampling. However, they carry potential procedural risks in premature and low birth weight neonates [1].

The CDC describes purulent sputum as a sample that contains more than or equal to 25 neutrophils and less than or equal to 10 squamous epithelial cells per low-power field on quantitative analysis, which corresponds to many, heavy, numerous, or 4+ neutrophils on semiquantitative reporting format. The threshold values described by the CDC for cultured specimens used in the microbiological diagnosis of VAP in older patients (not included in current guidelines for VAP in less than one year of age), depending on techniques, are ≥10^5^ CFU/mL for ETAs, ≥10^4^ CFU/mL for bronchoalveolar lavage, and ≥ 10^3 ^CFU/mL for protected specimen brushing on quantitative analysis, which corresponds to moderate, heavy, or numerous growths or 2+, 3+, or 4+ growth on semiquantitative reporting format [8,22]. When surveyors were asked what quantity of polymorphonuclear neutrophils (PMNs) on gram stain would be considered an infection rather than colonization or contamination, 68.5% reported the presence of moderate to large amounts of PMNs is regarded as an infection and can affect their decision to treat with antibiotics. This is contrary to microbiological results, where the same percentage (68.8%) of providers reported that their decision to treat with antibiotics does not depend on the burden of bacteria; instead, it relies on the type of bacteria. This could be because it is challenging to interpret respiratory culture results with certainty due to a lack of consensus among providers on differentiating colonization from pathological infection [23]. Another potential reason could be the lack of standardization in the laboratory reporting format of respiratory culture. This results in a gap in the provider’s knowledge about the cut-off value of cultures consistent with infection.

Chest X-ray is a mandatory criterion in the CDC VAP guidelines. However, its low diagnostic sensitivity and specificity in ventilated patients with underlying lung pathologies like chronic lung disease or RDS make it an unreliable tool. The inter-rater reliability of chest X-ray interpretation is also low [24]. In one study, only 64.5% of episodes met radiological criteria for VAP diagnosis based on chest X-ray [25]. Despite these inaccuracies, nearly 77% of respondents still choose to take a respiratory culture from patients with underlying lung disease who have infiltrates on chest X-rays and meet clinical criteria for VAP. For neonates, lung ultrasound (LU) is becoming an increasingly popular diagnostic tool for detecting lung pathologies. It has high inter-rater reliability and is highly accurate in diagnosing pneumonia in children [26]. Nora et al. have proposed a multi-parameter score that combines clinical, laboratory, microbiological, and LU data to diagnose VAP in neonates with underlying lung pathology. This score also leverages LU to monitor the response to antibiotic treatment, as the ideal duration for VAP treatment is still uncertain [25].

Participants in the survey needed to reach a consensus on classifying intubated patients with clinical symptoms of respiratory infection and those with positive respiratory culture results for a definitive diagnosis. The definition of VAT needs more clarity and consensus. VAT refers to a respiratory tract infection in an intubated mechanically ventilated patient with no radiological infiltrates. However, it is difficult to distinguish between VAT and VAP by identifying new or progressive infiltrates on CXR, especially for NICU patients where chest X-rays have certain limitations. Due to these difficulties in diagnosing VAP, in 2021, the National Healthcare Safety Network (NHSN) has transitioned to monitoring VAE in neonates, which are more broadly defined based on the increase in the fraction of inspired oxygen (FiO_2_) and/or mean airway pressure (MAP) after a period of stability or improvement on the ventilator [27]. NHSN criteria for VAE were developed for population-wide surveillance and were not intended to be used as a clinical diagnosis [1]. For this reason, as new diagnostic technologies become available, the formal definition of neonatal VAP may continue to evolve.

The optimal treatment approach for neonatal VAP remains to be determined. However, it is imperative to exercise caution when selecting antibiotic regimens given the correlation between dysbiosis and adverse outcomes such as necrotizing enterocolitis (NEC), BPD, retinopathy of prematurity, late-onset sepsis, systemic candidiasis, neurodevelopmental impairment, and death [28,29]. Empiric antibiotic regimens should be evaluated in the context of the local antibiogram, the patient’s clinical history, and respiratory culture results, which must be interpreted with care. Alriyami et al. recommend administering two broad-spectrum intravenous antibiotic combination therapies covering gram-positive and gram-negative organisms, including drug-resistant strains commonly found in nosocomial settings [1]. Vancomycin, in combination with gentamicin, exemplifies potential drug combinations. Third agents, such as antipseudomonal cephalosporins (e.g., cefepime or ceftazidime), may be necessary for severe or non-responsive patients. If aspiration pneumonia is a concern, antibiotics that are highly effective against anaerobes may be chosen, such as ampicillin-sulbactam, clindamycin, piperacillin-tazobactam, or meropenem. Antibiotic therapy should be tailored in cases where respiratory secretion analyses suggest a polymicrobial infection. Our survey also identified intravenous vancomycin and gentamicin as the most frequently prescribed empiric antibiotics. Intravenous cephalosporins were recognized as the third most common agent, followed by IV ampicillin and nafcillin.

A campaign against prolonged antibiotic therapy is currently underway as part of antibiotic stewardship. The initiative aims to ensure the judicious use of antibiotics in managing neonatal VAP to minimize the risk of antibiotic resistance and other adverse effects while optimizing therapeutic outcomes. For adult patients, the Infectious Disease Society of America and the American Thoracic Society strongly recommend a seven-day course of antibiotic therapy for VAP with modifications based on clinical course, labs, and radiological parameters [30]. However, there is no firm consensus on the duration of treatment for neonatal VAP. Studies have reported antibiotic courses ranging from five to 14 days [1]. Recent evidence favors shorter durations. Cantey et al. implemented a five-day antibiotic treatment course utilizing prospective surveillance and found that no infant required additional antibiotics for the same indication within 14 days of completing treatment [31].

Similarly, the neonatal Antimicrobial Stewardship Program (NEO-ASP) at Nationwide Children’s Hospital, Columbus, OH, and its seven affiliated NICUs recommended adherence to a five-day definitive antibiotic treatment course for “blood culture-negative” pneumonia. This intervention seemed safe in their prospective surveillance study as only 3% of pneumonia episodes relapsed 14 days after a five-day antibiotic course [32]. Goerens et al. proposed a three-part antibiotic stewardship program for infants with suspected VAP [9]. Decisions about whether to continue antibiotics are guided by clinical, laboratory, and microbiological re-evaluation after a trial of 48 to 72 hours of empiric therapy. Antibiotic treatment duration also varies among our survey participants, with more than half (55.5%) reporting treatment for a maximum of seven days, while 19.3% for five days, 11.4% for 10 days, and only 1.5% for 14 days. Due to a lack of agreement on the duration of treatment, further research is needed to determine the optimal course of treatment for neonatal VAP across different geographic centers and larger cohorts to provide clinical guidelines to providers. 

Alriyami et al. recommend early consultation with pediatric infectious disease specialists [1]. We found that 65.3% of providers would seek consultation from infectious disease specialists and 31.8% did not perceive the need, and only 1.18% would consult a pulmonologist. Given the potential complications of VAP, timely and appropriate consultation is pivotal for optimal outcomes.

There is an accumulating body of evidence advocating the efficacy of care bundles to prevent neonatal VAP. These bundles encompass a range of measures, including optimizing respiratory status with noninvasive respiratory support such as nasal continuous positive airway pressure (NCPAP), nasal intermittent positive pressure ventilation (NIPPV), bi-level positive pressure (BI-PAP), high flow nasal cannula (HFNC), and newer non-invasive ventilation strategies currently being studied including, nasal high-frequency ventilation (NHFV) and non-invasive neutrally adjusted ventilatory assist (NIV-NAVA), use of less invasive surfactant administration (LISA), minimally invasive surfactant therapy (MIST) techniques for surfactant administration, early planned extubation, antimicrobial stewardship, and infection control. Such efforts involve daily assessing the need for intubation and IMV, reducing the duration of IMV, using noninvasive respiratory support, avoiding reintubation, elevating the patient's head, timed mouth care, practicing proper hand hygiene, following isolation guidelines, and appropriately handling patient care equipment, sterile instruments, devices, and personal protective equipment [1,9]. Adhering to these measures has been linked with a reduced incidence of VAP and a significant decrease in antibiotic usage [9]. In the NICU setting, González et al. conducted a study on the implementation of a VAP prevention bundle, which resulted in a significant reduction in VAP rate from 12.89 to 1.31 episodes per 1000 ventilator days, a decrease in mortality from 21.3% to 13.2%, and ultimately a tendency toward clinical improvement [33]. However, our survey found that only slightly over half (53.5%) of participants have VAP prevention guidelines in their NICU. The potential explanation could be that neonatal VAP, a preventable HAI, has not received the same national attention among pediatric quality improvement networks as CLABSI due to the lack of a clear definition and gold standard diagnostic tests [34]. This survey study underscores the significance of adopting prevention care bundles to reduce the incidence of VAP and enhance patient outcomes. Therefore, it is recommended that healthcare facilities develop and implement multifaceted guidelines for VAP prevention in their NICUs.

Our survey shows a wide variation in the NICU provider’s approach toward investigating, diagnosing, treating, and preventing neonatal VAP. Despite the low overall response rate, the data we collected offers valuable insight into the current practices and opinions of American practicing neonatologists. Furthermore, it emphasizes the importance of developing and executing center-specific written protocols for VAP prevention through quality improvement initiatives. These measures will facilitate agreement among providers and guarantee improved patient outcomes.

Our study is subject to certain limitations, as the response to the current survey was voluntary, and there was potential for self-selection bias. The survey responses could also be affected by recall bias. The answers may reflect personal opinions rather than actual practice. We did not examine the potential reasons and explanations for these differences between providers.

## Conclusions

Although neonatal VAP is the second most common healthcare-associated infection in the NICU, there needs to be more information in the literature regarding workup and management. In this survey, it was demonstrated that there are inconsistencies in the investigation, diagnostic nomenclature, choice of antibiotic, and treatment duration for neonatal VAP across the country. Moreover, this study has revealed that NICU providers have varying knowledge about the policies for preventing VAP in their units. These findings highlight the need for further research to establish a clear definition and evidence-based criteria for VAP in the context of neonatal intensive care and, hence, decrease the knowledge gap. It is essential to focus on developing and implementing consensus-based VAP protocols to prevent, diagnose, and treat this condition to enhance outcomes for our vulnerable NICU population ultimately.

## Appendices

1. Have you obtained a respiratory culture in neonates on invasive mechanical ventilation in the last year as a part of the sepsis workup? Yes, No, Not sure

2. Do you perform routine surveillance respiratory culture in neonates on invasive mechanical ventilation? Yes, No, Not sure

2b. If yes, how often do you obtain surveillance respiratory culture? Daily, Every 48 hours, Weekly, Bi-weekly or more, As needed irrespective of time

3. In your opinion, how long should a neonate be on invasive mechanical ventilation before you obtain respiratory culture as part of sepsis work up? 0-2 days, 3-7 days, 8-14 days, >14 days, As needed irrespective of time

4. Do you routinely obtain respiratory culture as a potential source of CLABSI? Yes, No, Not sure

5. In which of the following circumstances do you consider obtaining respiratory culture in neonates with underlying diseases like RDS, BPD, lung edema, etc.? Please check all that apply. Temperature instability (fever or hypothermia), Increased respiratory rate or work of breathing, Change in character of tracheal secretions/purulence, Increased respiratory secretions/suctioning requirements, Increased FiO_2_ requirement, Worsening gas exchange, Increased infiltrates on chest X-ray, If others, please specify __________________________________

6. For those neonates with above underlying disease with chest X-ray showing infiltrates, when do you consider obtaining respiratory culture? Would not obtain a respiratory culture, Infiltrates on 1 chest X-ray, Infiltrates on 2 or more serial chest X-rays

7. Which of the following techniques do you use to collect specimens for respiratory culture in neonates Non-bronchoscopic bronchoalveolar lavage (NB-BAL) on invasive mechanical ventilation? Tracheal aspirate (ETA), Non-bronchoscopic bronchoalveolar lavage (NB-BAL), Non-bronchoscopic-protected specimen brush (NB-PSB), Bronchoscopic bronchoalveolar lavage (B-BAL), Do not obtain a respiratory culture

8. Do you always perform gram stain when obtaining a respiratory culture? Yes, No, Not sure

8b. If yes. Does amount of polymorphonuclear neutrophils (PMNs) on the gram stain affect your decision to treat patient with antibiotics? Yes, No, Not sure

8c. If yes, what quantity of polymorphonuclear neutrophils (PMNs) on the gram stain is more suggestive of infection rather than contamination or colonization? Rare or small amounts of WBCs, Moderate amount of WBCs, Large amounts of WBCs

9. Does bacterial burden on quantitative or semiquantitative analysis affect your decision to treat patient with antibiotics? Yes, No, Not sure

9b. If yes, what level of bacterial growth (depending on your lab reporting system) is more suggestive of infection rather than contamination or colonization? 1+ bacterial isolate, 2+ bacterial isolate, 3+ bacterial isolate, 4+ bacterial isolate or more, ≥10^1 cfu/mL bacterial isolate, ≥10^2 cfu/mL bacterial isolate, ≥10^3 cfu/mL bacterial isolate, ≥10^4 cfu/mL bacterial isolate or more

10. Does the type of the bacteria on respiratory culture affect your decision to treat patient? Yes, No, Not sure

11. In which of the following circumstances do you attribute bacterial growth to colonization rather than infection? Please check all that apply. Normal serum inflammatory markers and leukocytes, No or small amount of WBCs on gram stain, Normal respiratory flora on respiratory culture, Second-time positive respiratory culture with same organism after one course of treatment, Improvement in chest X-ray and respiratory status in 48-72 hours, If others, please specify: __________________________________ 

12. What is/are the most common organism(s) typically isolated from the respiratory culture in your practice? Please check all that apply. CONS, MRSA, Klebsiella, Enterobacter, Stenotrophomonas, Pseudomonas, E. Coli, Proteus, Streptococcus, Moraxella,  Hemophilus, Mycoplasma, Ureaplasma, If others, please specify: __________________________________

13. What diagnosis will you provide if the respiratory culture obtained by deep endotracheal aspirate comes back positive in presence of increased need for oxygen or ventilatory support and abnormal serum leukocytes or inflammatory markers in the setting of increased infiltrates on chest X-ray in neonates with underlying diseases like RDS, BPD, lung edema, etc. Ventilator-associated pneumonia (VAP), Ventilator-associated events (VAE), Ventilator-associated lower respiratory tract infections (VALRTIs), Ventilator-associated tracheobronchitis (VAT), Pneumonia, Tracheitis, If others please specify __________________________________

14. Which of the following antibiotics do you prescribe empirically pending respiratory culture results in a neonate ≥72 hours of age who has never got a respiratory culture before and been on invasive mechanical ventilator ≥48 hours? Please check all that apply. Ampicillin, Nafcillin, Gentamicin, Vancomycin, Cephalosporins, IV Tobramycin, Inhaled Tobramycin, Flouroquinolones, Amoxicillin, Bactrim, If others, Please specify: __________________________________ 

15. Which of the following antibiotics do you prescribe empirically pending respiratory culture result in a neonate ≥72 h of age who has never got a respiratory culture before, been on invasive mechanical ventilator ≥48 hours, and is unstable or very sick appearing? Please check all that apply. Ampicillin, Nafcillin, Gentamicin, Vancomycin, Cephalosporins, IV Tobramycin, Inhaled Tobramycin, Flouroquinolones, Amoxicillin, Bactrim, If others, Please specify: __________________________________ 

16. How long do you usually treat neonates with positive respiratory culture? 36 hours, 2 days, 5 days, 7 days, 10 days, 14 days, If others, please specify __________________________________

17. If needed, who do you usually consult for Infectious disease antibiotic(s) recommendation for treatment of Pulmonologist respiratory infection? None If others, please specify __________________________________

18. Do you believe that your colleagues practice differently than you on when to obtain respiratory culture? Yes, No, Not sure

19. Do you believe that your colleagues practice differently than you when prescribing antibiotics on results for respiratory culture? Yes, No, Not sure

20. Do you have any ventilator-associated pneumonia/events prevention guidelines along the lines of healthcare-associated infections (HAIs) in your practice? Yes, No, Not sure

21. What is your current position in General Pediatrician Neonatal-Perinatal Medicine? Fellow Neonatologist Advanced Practice Provider, If others, please specify __________________________________

22. What is the size of your NICU? Less than 10 beds, 10-20 beds, 20-30 beds, More than 30 beds

23. What is the level of your NICU? Level I, Level II, Level III, Level IV

24. What is your primary practice setting? Both fellowship and residency training, Residency training only, Community hospital without fellowship and residency training, Private practice If others, please specify  __________________________________

25. What region of the United States do you work in? Southwest, Southeast, Midwest, West, Northeast
